# Ultra‐Processed food intake and risk of *Helicobacter pylori* infection: A case–control study

**DOI:** 10.1002/fsn3.4152

**Published:** 2024-04-01

**Authors:** Zohreh Ebrahimi, Zainab Shateri, Mehran Nouri, Masoumeh Khalighi Sikaroudi, Mohsen Masoodi, Farzad Shidfar, Mahdi Hejazi

**Affiliations:** ^1^ Department of Nutrition, School of Public Health Iran University of Medical Sciences Tehran Iran; ^2^ Student Research Committee Ahvaz Jundishapur University of Medical Sciences Ahvaz Iran; ^3^ Infectious Diseases and Tropical Medicine Research Center, Health Research Institute Babol University of Medical Sciences Babol Iran; ^4^ Department of Clinical Nutrition, School of Nutritional Sciences and Dietetics Tehran University of Medical Sciences Tehran Iran; ^5^ Colorectal Research Center Iran University of Medical Sciences Tehran Iran; ^6^ Nutritional Sciences Research Center Iran University of Medical Sciences Tehran Iran

**Keywords:** carbohydrates, fat, *Helicobacter pylori*, salt, UPFs

## Abstract

The components in our food are known as one of the important risk factors for the development of *Helicobacter pylori* (*H. pylori*) infection. A balanced diet, rich in fruits and vegetables, and free of fat, sugar, and salt, might protect people from the consequences of *H. pylori* infection. Therefore, the purpose of this study was to investigate the associations between ultra‐processed foods (UPFs) intake and the risk of *H. pylori* infection. The case–control study was conducted to assess the intake of UPFs in patients with *H. pylori* infection compared with healthy individuals. The dietary data of the contributors were collected by a validated food frequency questionnaire (FFQ). To estimate the UPFs intake, the classification of the NOVA food group was utilized. The associations of intake UPFs with *H. pylori* infection were assessed using binary logistic regression. Finally, dietary data of 150 cases and 302 controls (mean age: 39.5 ± 10.95 years) were analyzed. UPFs intake was associated with higher risk of *H. pylori* infection (odds ratio (OR) = 1.71; 95% confidence interval (CI): 1.05, 2.79). The association remained constant after adjustment for age, body mass index (BMI), sex, energy intake, physical activity, smoking, and alcohol status (OR = 2.17; 95% CI: 1.22, 3.86). Our data declare that UPFs consumption could have a role in increasing the likelihood of the risk of *H. pylori* infection. To confirm the current findings, prospective studies are suggested.

## Graphical abstract

Ultra‐processed foods (UPFs) as a continuous variable were directly associated with *Helicobacter pylori* (*H. pylori*) infection, even after adjustment for age, body mass index (BMI), sex, energy intake, physical activity, smoking, and alcohol status.

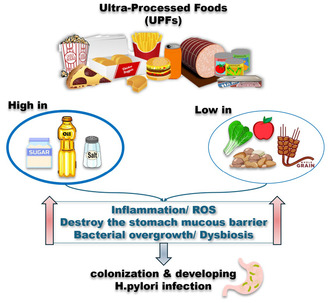

## 1 INTRODUCTION

*Helicobacter pylori* (*H. pylori*) is one of the most widespread reasons of earnest chronic bacterial infections (Aumpan et al., 2023) and it is known as the main agent of chronic gastritis, peptic ulcer, and primary gastric lymphoma (Bardazzi et al., 2020), that eight out of ten cases of gastric cancer have been assigned to it (de Martel et al., 2020). The varied pathologies ascribed to *H. pylori* infection are caused by the complex interplay of bacterial, host genetics, and environmental agents (Amieva & El–Omar, 2008; Malfertheiner et al., 2023). Along with other modifiable environmental factors, nutrition and nutritional status play an important role in *H. pylori* infection (Öztekin et al., 2021).

During the past years, the consumption of ultra‐processed foods (UPFs) has increased dramatically worldwide, replacing healthy dietary patterns (nuts, legumes, vegetables, and fruits) (Leo et al., 2020). These foods are placed in the fourth category of Nova system in terms of the physical, biological, and chemical characteristics. The categories of Nova system include: 1. Fresh and minimally processed foods, without adding new substances to extend shelf‐life (for example: milled cereals, meats, eggs, milk, vegetables, nuts, and seeds). 2. Cooking ingredients for use in food preparation (such as salt, vegetable oils, vinegar, butter, and sugar). 3. Processed food that is generally prepared using combinations of culinary ingredients in categories 1 and 2 (such as canned fish or beans). 4. Ultra‐processed foods that include industrially formulated food, usually prepared with five or more ingredients from the category 1 or 2 and food additives through a series of industrial processes (such as soft drinks; chocolate, candies, ice cream, biscuits, and cakes; packaged leaves of bread; nuggets and sticks, margarine, and pastries; preprepared food dishes) (Monteiro et al., 2018). UPFs are rich in fat (saturated and trans‐fatty acids), sugar, calorie, and salt but poor in protein, fiber, micronutrients, and other bioactive compounds and include lower nutritional quality foods, compared to fresh or homemade foods (Monteiro et al., 2018).

These foods and their components have been indicated to negatively influence gut microbiota, systemic inflammation, and body weight, raising concerns relating to their long‐time impacts on health (Chassaing et al., 2015; Srour et al., 2022). Possible mechanisms of their association with health status are food composition and their “processing.” UPFs are typically nutritionally inappropriate due to their composition (Martini et al., 2021). For example, UPFs contain large amounts of salt, which helps to increase the colonization of *H. pylori* by destroying the mucous barrier of the stomach, and in the long term leads to gastritis resulting from *H. pylori* (Caruso & Fucci, 1990; Fox et al., 1999). It seems that high contents of carbohydrates in UPFs can play a role in the pathogenesis of *H. pylori* infection by delaying gastric emptying and disrupting gastrin secretion (Mourot et al., 1988). Moreover, evidence shows that high consumption of UPFs is potentially related to the high rate of obesity (Cordova et al., 2021), which has been identified as a factor for increasing the risk of *H. pylori* infection (Suki et al., 2018; Xu et al., 2014). UPFs consumption can also play a role in creating an inflammatory state through different mechanisms. UPFs may create an environment that is more conducive to the growth of *H. pylori*. Moreover, this diet may enhance oxidative stress and inflammation, which can disturb the immune response to *H. pylori* (Tristan Asensi et al., 2023). Consequently, consuming high amounts of UPFs occasionally leads to the substitution of foods that are the basis of a healthy and balanced diet such as fruits and vegetables, which are associated with anti‐inflammatory effects due to the presence of multiple compounds (Tristan Asensi et al., 2023). Considering the widespread prevalence of *Helicobacter* (in the world and Iran) (Hooi et al., 2017; Mezmale et al., 2020) and its role in causing digestive problems and gut cancers and the adverse consequences of consuming UPFs in the gastrointestinal tract, it seems that investigating the possible association between UPFs intake and the risk of *H. pylori* infection can become very necessary. Therefore, an appropriate and balanced diet, rich in fruits and vegetables, and free of fat, sugar, and salt, might protect people against the consequences of *H. pylori* infection (Fox et al., 1999; Mard et al., 2014; Sohouli et al., 2021; Xia et al., 2016). To the best of our knowledge, the present study is the first study that was designed and implemented to assess the relationship between UPFs and *Helicobacter pylori* infection.

## 2 METHODS

### 2.1 Study design and participants

This case–control study was done on 150 patients with *H. pylori* infection and 302 healthy subjects who were referred to the Rasoul‐e‐Akram Hospital, Tehran, Iran between June 2021 and November 2021. The participants in the case group (*H. pylori* positive) were recruited from among patients who were in the active phase of infection and were not treated before participating in this study. The control group were subjects who had no infection with *H. pylori* according to the diagnostic methods. The persons who had *H. pylori* previously and were treated or who are resistant to treatment were not included in the control group and were excluded from the study. We excluded patients who had BMI <18.5 or > 35 kg/m^2^, malignant or inflammatory disease, a medical history of psychosis or memory disturbances, diabetes, liver, and cardiovascular diseases, following special diet (vegan, ketogenic, fasting, etc.), pregnant, lactating, lack of literacy to fill out the questionnaire, and consuming any nutritional supplements (e.g., omega‐3 fatty acids) during recent 6 months. Moreover, after collecting data, persons who had reported energy intake out of 800–4200 kcal/d were excluded (Fung et al., 2002).

The study protocol was approved by the Ethics Committee of the Iran University of Medical Sciences (IR.IUMS.REC 1396.32632). The written informed consent form was collected from each participant.

### 2.2 Anthropometric assessment

Anthropometric measurements were performed by a trained nutritionist. Weight was assessed with least clothing, without shoes, using a digital scale (Seca 807) (100 g precision). Standing height assessment was done without shoes by a stadiometer (Seca 206) (0.5 cm precision). Waist circumference (WC) was measured using a flexible tape at the midpoint between the last rib and the iliac crest at minimal respiration (0.5 cm precision).

### 2.3 Assessment of other variables

Information on age, gender (male/female), smoking (yes/no), and alcohol consumption (yes/no) was collected with a self‐administered questionnaire. We used the International Physical Activity Questionnaire (IPAQ) to measure the activity of participants (Craig et al., 2003). All data of the IPAQ were expressed as metabolic equivalents per week (MET‐minute/week).

### 2.4 Diagnosis tools

*Helicobacter pylori* infection should be distinguished by the serological antibody testing (Immunoglobulin G (IgG) and Immunoglobulin A (IgA)) in blood samples, stool antigen testing in fecal matter samples, urea breath test (UBT), endoscopy, and gastric biopsy.

### 2.5 Dietary assessment

Dietary intakes of the contributors were evaluated by a 168‐item food frequency questionnaire (FFQ) that was already validated in the Iranian population (Esfahani et al., 2010). Two experienced interviewers completed the FFQ, and participants were asked to express their dietary intake in the form of day, week, month, or year.

The NOVA food group classification was utilized to determine the UPFs (Monteiro et al., 2018). Food and beverages from FFQ (biscuits, pastries, cakes, confectionary, some breads, meat products, sauces, soft drinks, sugar‐sweetened milk, ice cream, frozen yogurts, french fries, pizza, packaged salty snacks, fruit drinks, dressing and gravies, margarine and other spreads) were known as UPFs (Lane et al., 2022). To characterize the portion of each subgroup of UPFs to overall UPFs intake, the average daily intake of each of UPFs subgroups (dairy products, nondairy beverages, margarine and sauces, cakes and cookies, chips and snacks, fast food, and meat, sweet, and others) was divided by the total UPFs daily intake and then multiplied by 100.

### 2.6 Statistical analysis

The normality of the data was assessed using the Kolmogorov–Smirnov test and histogram chart. Baseline data and dietary intakes of participants were stated as mean ± SD (for quantitative variables with normal distribution) and median (for quantitative variables with skewed distribution). Comparison of the continuous and categorical variables between two groups was done using independent sample *t*‐test and chi square, respectively. Also, we used binary logistic regression in different adjusted models to obtain odds ratio (OR) and 95% confidence interval (CI) for the relationship between UPFs and *Helicobacter pylori* infection. All analyses were done by the SPSS software (version 21). *P*‐values less than .05 were considered significant levels.

## 3 RESULTS

**FIGURE 1:**
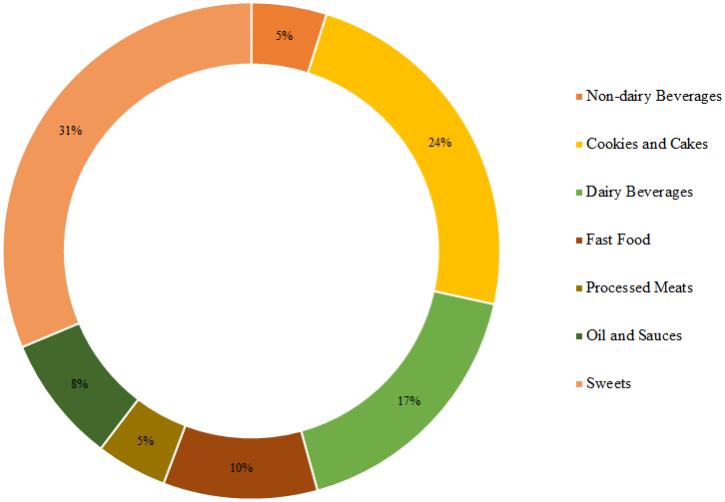
The contribution (%) of total UPFs subgroups consumption.

**FIGURE 2:**
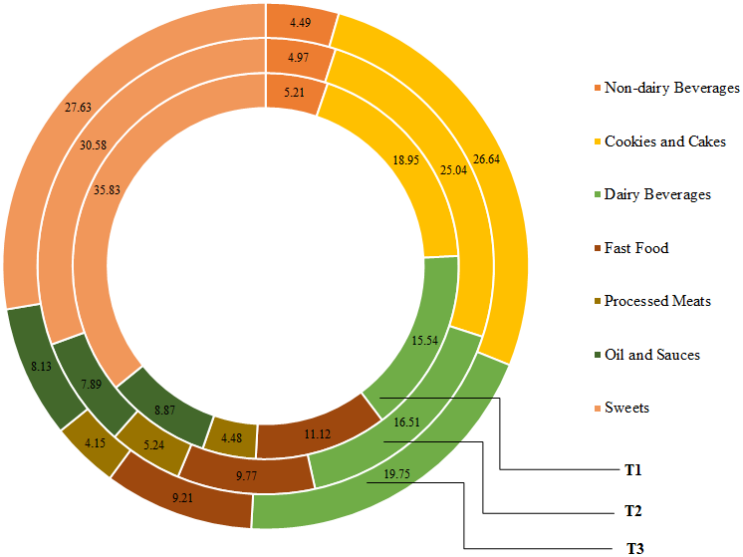
The contribution of UPFs subgroups consumption across UPFs tertile. T1: tertile 1. T2: tertile 2. T3: tertile 3.

One hundred fifty cases and 302 controls with complete data and a mean age of 32.31 ± 10.96 years (61.7% were male) were included in the statistical analysis. The demographic characteristics of case and control are represented in Table 1. Compared with those in the control groups, the case groups were significantly older, had higher BMI, had higher waist circumference, and were more likely to be current smokers. No other significant difference was observed in this regard. As we show in Table 2, individuals with *H. pylori* had a higher intake of energy, protein, carbohydrate, nondairy beverages, fast foods, oils, and sauces, and a lower intake of total fat, processed meats, and sweets compared to controls. The contribution (%) of total UPFs subgroups consumption is shown in Figure 1. Also, the contribution of UPFs subgroups consumption across UPFs tertile is represented in Figure 2.

**TABLE 1:** The features of case and control participants.

Variables	Cases (150)	Controls (302)	*p*‐value
Age (year)^b^	42.0 ± 13.5	37.1 ± 8.4	<.001
BMI (kg/m^2^)^b^	28.1 ± 6.6	24.8 ± 3.2	<.001
Waist circumference (cm)^b^	108.8 ± 13.6	96.5 ± 6.3	<.001
Male, *N* (%)^a^	48 (32.0)	135 (44.7)	.011
Current smokers, *N* (%)^a^	11 (7.3)	8 (2.6)	.025
Alcohol consumption, *N* (%)^a^	16 (10.7)	21 (7.0)	.203
*Helicobacter pylor*i diagnosis, *N* (%)^a^			.167
Endoscopic biopsy	51 (34.0)	75 (24.8)	
Urea breath test	30 (20.0)	81 (26.8)	
Serum immunoglobulin	37 (24.7)	80 (26.5)	
Stool antibody	32 (21.3)	66 (21.9)	
Physical activity, *N* (%)^a^			.484
Low	114 (76.0)	220 (72.8)	
Moderate	29 (19.3)	59 (19.5)	
High	7 (4.7)	23 (7.6)	

*Note*: Data are presented as mean ± SD for continuous and percentage for categorical variables.

Abbreviation: BMI, Body mass index.

^a^ Using chi‐square test for categorical variables.

^b^ Using independent samples *T*‐test for normal continuous variables.

**TABLE 2:** Dietary intake among the case and control participants.

Variables	Cases (150)	Controls (302)	*p*‐value
Energy (kcal/day)^b^	2590.7 (932.8)	2246.9 (854.9)	<.001
Protein (energy %)^a^	14.3 ± 2.2	13.5 ± 2.3	<.001
Carbohydrate (energy %)^b^	60.3 (7.0)	56.9 (9.5)	<.001
Total fat (energy %)^b^	29.0 (17.2)	31.4 (9.1)	.018
UPFs (energy %)^b^	9.8 (7.2)	8.7 (7.5)	.072
Nondairy beverages (UPFs energy %)^b^	4.9 (6.2)	2.5 (4.5)	.034
Cookie and cakes (UPFs energy %)^b^	20.0 (23.5)	18.2 (25.1)	.383
Dairy beverages (UPFs energy %)^b^	12.8 (15.9)	13.0 (18.3)	.238
Fast foods (UPFs energy %)^b^	6.9 (17.9)	5.2 (9.1)	.005
Processed meats (UPFs energy %)^b^	1.1 (4.3)	3.6 (6.8)	<.001
Oils and sauces (UPFs energy %)^b^	10.4 (16.1)	3.3 (6.6)	<.001
Sweets (UPFs energy %)^b^	22.6 (19.0)	31.9 (25.6)	<.001

*Note*: Data are presented as mean ± SD or median (interquartile range (IQR)) for continuous variables.

Abbreviation: UPFs, Ultra‐processed foods.

^a^ Using independent samples *T*‐test for normal continuous variables.

^b^ Using Mann–Whitney for abnormal continuous variables.

The association between UPFs and *Helicobacter pylori* is shown in Table 3. In comparison to the first tertile, we observed a significant higher odds of *H. pylori* infection in the third tertile of UPFs in the crude model (OR = 1.71; 95% CI: 1.05, 2.79). After adjusting the role of age, BMI, and sex in the second model, a higher odds of *H. pylori* infection still were seen in the third tertile in comparison to the first one (OR = 2.21; 95% CI: 1.29, 3.77). In comparison to the first tertile in the third model, after adjusting the role of potential confounders (age, BMI, sex, energy intake, physical activity, smoking, and alcohol status), a significant association was seen in the second and last tertile (T_2_: OR = 1.83; 95% CI: 1.02, 3.28 and T_3_: OR = 2.17; 95% CI: 1.22, 3.86).

**TABLE 3:** Association between tertiles of ultra‐processed foods and risk of *Helicobacter pylori* infection.

Tertiles of ultra‐processed foods	Case (*N*)	Control (*N*)	Model 1 OR (95% CI)	Model 2 OR (95% CI)	Model 3 OR (95% CI)
T_1_ (≤6.86% energy)	40	110	Ref.	Ref.	Ref.
T_2_ (6.87–11.48% energy)	52	99	1.44 (0.88–2.36)	1.66 (0.97–2.84)	1.83 (1.02–3.28)
T_3_ (≥11.49% energy)^a^	58	93	1.71 (1.05–2.79)	2.21 (1.29–3.77)	2.17 (1.22–3.86)
p*			.031	.004	.012

*Note*: Data are presented as OR (95% CI). The T1 tertile was considered as reference.

Model 1: crude model.

Model 2: adjusted for age, BMI, and sex.

Model 3: adjusted for age, BMI, sex, energy intake, physical activity, smoking, and alcohol status.

^a^ The third tertile of UPFs was considered as high UPFs intake.

*p for trend: obtained from binary logistic regression.

## 4 DISCUSSION

The present study's findings showed that the consumption of UPF, which was associated with a high intake of sweets and cakes, nondairy drinks, dairy drinks, processed meats, and fast foods, was positively associated with the chance of developing *H. pylori* infection. According to our results, higher UPFs intake leads to 2.17 times increased odds of *H. pylori* infection.

A positive and significant relationship between UPFs consumption and irritable bowel syndrome and dyspepsia (Schnabel et al., 2018), weight gain (Hall et al., 2019), cardiovascular disease (Srour et al., 2019), type 2 diabetes mellitus (Srour et al., 2020), and inflammatory diseases (Srour et al., 2022) has been reported. However, so far no study has investigated the association between UPFs and *H. pylori*. Therefore, in this study, we investigated this relationship that based on our results, there was a significant relationship between UPFs and *H. pylori* infection.

Ultra‐processed foods (UPFs) are usually high in fat, sugar, salt, and energy and low in minerals, fiber, protein, and vitamins (Monteiro et al., 2019). Also, UPFs contain ingredients used infrequently or in small amounts in cooking, such as high fructose corn syrup, hydrogenated oil, flavors, colors, artificial sweeteners, and maltodextrin, which are used with the purpose of making food tasty (Elizabeth et al., 2020).

Possible mechanisms of UPFs association with *H. pylori* infection may be the components contained and their “processing.” In fact, UPFs are typically nutritionally inappropriate due to their compositions. For example, UPFs are rich in salt, and high amounts of salt consumption over time can destroy the mucous barrier of the stomach, which helps to increase the colonization of *H. pylori*, which in the long term causes gastritis caused by *H. pylori* infection (Caruso & Fucci, 1990; Fox et al., 1999). In this regard, Shu et al. indicated that a higher salt intake was associated with a higher risk of *H. pylori* infection (Shu et al., 2019). In an ecological study by Beevers et al., the findings showed a positive relationship between salt intake and the risk of *H. pylori* (Beevers et al., 2004). It seems that high content of carbohydrates (e.g., soft drinks) in UPFs may play a role in the pathogenesis of *H. pylori* infection by increasing urea influx and ammonia production by the bacteria due to prolonging the periods of gastric acidification (Nseir et al., 2012).

Ultra‐processed foods (UPFs) play a role in many chronic inflammatory diseases, such as metabolic syndrome and inflammatory bowel disease (Srour et al., 2022). Also, it has been shown that inflammation can affect the occurrence of bacterial infections. Furthermore, the presence of inflammation is a favorable condition for the growth of *H. pylori* (Meduri, 2001). In a cross‐sectional study, it was observed that UPFs consumption in adolescents was associated with an increase in inflammatory markers, such as C‐reactive protein (CRP) and interleukin (IL)‐8 (dos Santos et al., 2022). The role of UPFs in causing inflammation can be attributed to the presence of its high saturated fatty acid (SFA) and fructose. In a systematic review study, it has been reported that the consumption of SFA was associated with an increase in high sensitivity‐CRP (hs‐CRP) (Santos et al., 2013). Also, the role of fructose in increasing inflammatory biomarkers has been indicated (Aeberli et al., 2011; Cox et al., 2011).

Also, the studies revealed that people with *H. pylori* received more fast foods and oils. In addition to the content of fast foods (sodium, sugar, SFA, and trans‐fatty acids), they can be related to the risk of contracting *Helicobacter pylori* by unsuitable food handling, poor sanitation practices, and its transmission from person to person (Kassid et al., 2022; Mhaskar et al., 2013). Finding from a case–control study showed that the consumption of restaurant foods is a risk factor for developing *H. pylori* (Mhaskar et al., 2013). Also, in a study by Kassid et al., a significant relationship was seen between fast food consumption and an increased incidence of *H. pylori* (Kassid et al., 2022). In another study in 2002, it was indicated that fast food consumption was related to an increased incidence of *H. pylori* (Kyriazanos et al., 2002).

The current study illustrated that people with *H. pylori* received more energy and had more body weight and waist circumference. UPFs, such as potato chips, sugar‐sweetened beverages, sweets, desserts, and fried foods, are associated with weight gain (Crimarco et al., 2021; Elizabeth et al., 2020; Pereira et al., 2005). A positive linear correlation between *H. pylori* and BMI has been detected (Xu et al., 2014). Also, a study by Suki et al. showed a positive relationship between BMI and *H. pylori* (Suki et al., 2018). Our findings also illustrated that people with *H. pylori* have significantly greater weight than those with *H. pylori*‐negative people. Due to the study's cross‐sectional nature, the cause‐and‐effect relationship between *H. pylori* and BMI cannot be answered. However, some studies have demonstrated that obesity in both animals and humans may be linked to several microbes, including viruses, parasites, and bacteria (Hegde & Dhurandhar, 2013; Pasarica & Dhurandhar, 2007). Increasing BMI can cause changes in adaptive and innate immunity (Marti et al., 2001). These immunological changes may create a favorable immune environment for *H. pylori* (Xu et al., 2014).

A study by Mard et al. showed that lower consumption of fruits and vegetables and higher consumption of fast food increase the chance of acquiring *H. pylori* infection (Mard et al., 2014). A recent research clearly shows how persons who consume more UPFs have less fruit and vegetable consumption (Dinu et al., 2022). A healthy diet contains many compounds with anti‐inflammatory effects. Dietary antioxidants predominantly from fruits and vegetables can provide a balanced defense mechanism against the harmful effects of reactive oxygen species (ROS). Antioxidants inhibit bacterial growth by reducing reactive oxygen species and inflammations caused by *H. pylori* (YaoBin et al., 2019). In addition, low fruit and vegetable consumption results in a low dietary fiber intake, which disturbs the intestinal microbiota, and increases intestinal permeability and pro‐inflammatory response (Aguayo‐Patrón et al., 2017). Intestinal microbiota plays a role in strengthening the immune system and preventing the entry of pathogens by providing nutrients produced from fatty acids and vitamins (Oh et al., 2016). Changes in gut microbiota can interfere in *H. pylori*‐related immune responses (Chen et al., 2021). Therefore, consumption of UPFs can increase the chance of developing *H. pylori* by changing the intestinal microbiota and thus changing the function of the immune system.

The present study was the first to examine the relationship between UPFs consumption and the chance of *H. pylori* infection. Also, we used a reliable and valid questionnaire to collect the data. In addition, controlling many confounding factors to achieve more correct results was one of the strengths of the present study. One of the study's limitations is the lack of causal relationships due to the nature of the study. Also, using the FFQ questionnaire was one of the other limitations of the present study because the participants might have difficulty in remembering the type and amount of consumed foods. However, using this questionnaire in epidemiological studies is one of the best methods to collect dietary data.

## 5 CONCLUSION

In the present study, higher UPFs consumption was associated with a higher chance of getting affected by *H. pylori*. Also, the findings indicated increased energy intake and weight gain with receiving UPFs. In these people, the chance of acquiring *H. pylori* infection was also higher. More studies are needed to determine the exact mechanisms by which UPFs can increase the likelihood of developing *H. pylori*. Also, more studies are required to confirm the current study's findings.

## AUTHOR CONTRIBUTIONS

**Zohreh Ebrahimi:** Data curation (equal); formal analysis (equal); methodology (equal); resources (equal); software (equal); supervision (equal); validation (equal); writing – original draft (equal); writing – review and editing (equal). **Zainab Shateri:** Methodology (equal); writing – original draft (equal); writing – review and editing (equal). **Mehran Nouri:** Data curation (equal); investigation (equal); methodology (equal). **Masoumeh Khalighi Sikaroudi:** Contribution in sampling. **Mohsen Masoodi:** Data curation (equal); methodology (equal); writing – review and editing (equal). **Farzad Shidfar:** Conceptualization (equal); data curation (equal); formal analysis (equal); investigation (equal); methodology (equal); visualization (equal); writing – original draft (equal); writing – review and editing (equal). **Mahdi Hejazi:** Data curation (equal); writing – review and editing (equal).

## FUNDING INFORMATION

This research was not funded by any research center.

## CONFLICT OF INTEREST STATEMENT

The authors declared that there were no conflicts of interest.

## Data Availability

The datasets used or analyses during the current study are available from the corresponding author on reasonable request.

## References

[fsn34152-bib-0001] Aeberli, I. , Gerber, P. A. , Hochuli, M. , Kohler, S. , Haile, S. R. , Gouni‐Berthold, I. , Berthold, H. K. , Spinas, G. A. , & Berneis, K. (2011). Low to moderate sugar‐sweetened beverage consumption impairs glucose and lipid metabolism and promotes inflammation in healthy young men: A randomized controlled trial. The American Journal of Clinical Nutrition, 94(2), 479–485. 21677052 10.3945/ajcn.111.013540

[fsn34152-bib-0002] Aguayo‐Patrón, S. V. , Ana, M. , & de la Barca, C. (2017). Old fashioned vs. ultra‐processed‐based current diets: Possible implication in the increased susceptibility to type 1 diabetes and celiac disease in childhood. Food, 6(11), 100. 10.3390/foods6110100 PMC5704144 29140275

[fsn34152-bib-0003] Amieva, M. R. , & El–Omar, E. M. (2008). Host‐bacterial interactions in helicobacter pylori infection. Gastroenterology, 134(1), 306–323. 18166359 10.1053/j.gastro.2007.11.009

[fsn34152-bib-0004] Asensi, T. , Marta, A. N. , Sofi, F. , & Dinu, M. (2023). Low‐grade inflammation and ultra‐processed foods consumption: A review. Nutrients, 15(6), 1546. 36986276 10.3390/nu15061546 PMC10058108

[fsn34152-bib-0005] Aumpan, N. , Mahachai, V. , & Vilaichone, R.‐k. (2023). Management of Helicobacter pylori infection. JGH Open, 7(1), 3–15. 36660052 10.1002/jgh3.12843 PMC9840198

[fsn34152-bib-0006] Bardazzi, F. , Magnano, M. , Fiorini, G. , Vaira, D. , Odorici, G. , Bertusi, G. , & Patrizi, A. (2020). Helicobacter pylori infection in psoriatic patients during biological therapy. Italian Journal of Dermatology and Venereology, 156(5), 570–574. 10.23736/S2784-8671.19.06410-1 32041937

[fsn34152-bib-0007] Beevers, D. G. , Lip, G. Y. H. , & Blann, A. D. (2004). Salt intake and helicobacter pylori infection. Journal of Hypertension, 22(8), 1475–1477. 15257168 10.1097/01.hjh.0000133736.77866.77

[fsn34152-bib-0008] Caruso, M. L. , & Fucci, L. (1990). Histological identification of helicobacter pylori in early and advanced gastric cancer: To the editor. Journal of Clinical Gastroenterology, 12(5), 601–602. 2103734

[fsn34152-bib-0009] Chassaing, B. , Koren, O. , Goodrich, J. K. , Poole, A. C. , Srinivasan, S. , Ley, R. E. , & Gewirtz, A. T. (2015). Dietary emulsifiers impact the mouse gut microbiota promoting colitis and metabolic syndrome. Nature, 519(7541), 92–96. 25731162 10.1038/nature14232 PMC4910713

[fsn34152-bib-0010] Chen, C.‐C. , Liou, J.‐M. , Lee, Y.‐C. , Hong, T.‐C. , El‐Omar, E. M. , & Ming‐Shiang, W. (2021). The interplay between helicobacter pylori and gastrointestinal microbiota. Gut Microbes, 13(1), 1909459. 33938378 10.1080/19490976.2021.1909459 PMC8096336

[fsn34152-bib-0011] Cordova, R. , Kliemann, N. , Huybrechts, I. , Rauber, F. , Vamos, E. P. , Levy, R. B. , Wagner, K.‐H. , Viallon, V. , Casagrande, C. , & Nicolas, G. (2021). Consumption of ultra‐processed foods associated with weight gain and obesity in adults: A multi‐national cohort study. Clinical Nutrition, 40(9), 5079–5088. 34455267 10.1016/j.clnu.2021.08.009

[fsn34152-bib-0012] Cox, C. L. , Stanhope, K. L. , Schwarz, J. M. , Graham, J. L. , Hatcher, B. , Griffen, S. C. , Bremer, A. A. , Berglund, L. , McGahan, J. P. , & Keim, N. L. (2011). Circulating concentrations of monocyte chemoattractant protein‐1, plasminogen activator inhibitor‐1, and soluble leukocyte adhesion molecule‐1 in overweight/obese men and women consuming fructose‐or glucose‐sweetened beverages for 10 weeks. The Journal of Clinical Endocrinology & Metabolism, 96(12), E2034–E2038. 21956423 10.1210/jc.2011-1050 PMC3232623

[fsn34152-bib-0013] Craig, C. L. , Marshall, A. L. , Sjöström, M. , Bauman, A. E. , Booth, M. L. , Ainsworth, B. E. , Michael Pratt, U. L. F. , Ekelund, A. Y. , & Sallis, J. F. (2003). International physical activity questionnaire: 12‐country reliability and validity. Medicine & Science in Sports & Exercise, 35(8), 1381–1395. 12900694 10.1249/01.MSS.0000078924.61453.FB

[fsn34152-bib-0014] Crimarco, A. , Landry, M. J. , & Gardner, C. D. (2021). Ultra‐processed foods, weight gain, and co‐morbidity risk. Current Obesity Reports, 11(3), 80–92. 34677812 10.1007/s13679-021-00460-y PMC8532572

[fsn34152-bib-0015] de Martel, C. , Georges, D. , Bray, F. , Ferlay, J. , & Clifford, G. M. (2020). Global burden of cancer attributable to infections in 2018: A worldwide incidence analysis. The Lancet Global Health, 8(2), e180–e190. 31862245 10.1016/S2214-109X(19)30488-7

[fsn34152-bib-0016] Dinu, M. , Asensi, M. T. , Pagliai, G. , Lotti, S. , Martini, D. , Colombini, B. , & Sofi, F. (2022). Consumption of ultra‐processed foods is inversely associated with adherence to the Mediterranean diet: A cross‐sectional study. Nutrients, 14(10), 2073. 35631214 10.3390/nu14102073 PMC9147239

[fsn34152-bib-0017] dos Santos, M. , Márcia, G. , da Cunha França, A. K. T. , de Almeida Fonseca Viola, P. C. , de Carvalho, C. A. , Marques, K. D. S. , Santos, A. M. D. , Batalha, M. A. , de Alencar Alves, J. D. , & Ribeiro, C. C. C. (2022). Intake of ultra‐processed foods is associated with inflammatory markers in Brazilian adolescents. Public Health Nutrition, 25(3), 591–599. 34726140 10.1017/S1368980021004523 PMC9991817

[fsn34152-bib-0018] Elizabeth, L. , Machado, P. , Zinöcker, M. , Baker, P. , & Lawrence, M. (2020). Ultra‐processed foods and health outcomes: A narrative review. Nutrients, 12(7), 1955. 32630022 10.3390/nu12071955 PMC7399967

[fsn34152-bib-0019] Esfahani, F. H. , Asghari, G. , Mirmiran, P. , & Azizi, F. (2010). Reproducibility and relative validity of food group intake in a food frequency questionnaire developed for the Tehran lipid and glucose study. Journal of Epidemiology, 20(2), 150–158. 20154450 10.2188/jea.JE20090083 PMC3900814

[fsn34152-bib-0020] Fox, J. G. , Dangler, C. A. , Taylor, N. S. , King, A. , Koh, T. J. , & Wang, T. C. (1999). High‐salt diet induces gastric epithelial hyperplasia and parietal cell loss, and enhances helicobacter pylori colonization in C57BL/6 mice. Cancer Research, 59(19), 4823–4828. 10519391

[fsn34152-bib-0021] Fung, T. T. , Hu, F. B. , Pereira, M. A. , Liu, S. , Stampfer, M. J. , Colditz, G. A. , & Willett, W. C. (2002). Whole‐grain intake and the risk of type 2 diabetes: A prospective study in men. The American Journal of Clinical Nutrition, 76(3), 535–540. 12197996 10.1093/ajcn/76.3.535

[fsn34152-bib-0022] Hall, K. D. , Ayuketah, A. , Brychta, R. , Cai, H. , Cassimatis, T. , Chen, K. Y. , Chung, S. T. , Costa, E. , Courville, A. , & Darcey, V. (2019). Ultra‐processed diets cause excess calorie intake and weight gain: An inpatient randomized controlled trial of ad libitum food intake. Cell Metabolism, 30(1), 67–77. 31105044 10.1016/j.cmet.2019.05.008 PMC7946062

[fsn34152-bib-0023] Hegde, V. , & Dhurandhar, N. V. (2013). Microbes and obesity—Interrelationship between infection, adipose tissue and the immune system. Clinical Microbiology and Infection, 19(4), 314–320. 23506525 10.1111/1469-0691.12157

[fsn34152-bib-0024] Hooi, J. K. Y. , Lai, W. Y. , Ng, W. K. , Suen, M. M. Y. , Underwood, F. E. , Tanyingoh, D. , Malfertheiner, P. , Graham, D. Y. , Wong, V. W. S. , & Wu, J. C. Y. (2017). Global prevalence of helicobacter pylori infection: Systematic review and meta‐analysis. Gastroenterology, 153(2), 420–429. 28456631 10.1053/j.gastro.2017.04.022

[fsn34152-bib-0025] Kassid, O. M. , Alhashimi, R. A. H. , Alhelfi, H. S. Q. , & Alsaad, R. K. A. (2022). Prevalence and risk factors of helicobacter pylori infection in misan, Iraq: A cross‐sectional screening study using stool antigen test. Journal of Medicinal and Chemical Sciences, 5(7), 1177–1182.

[fsn34152-bib-0026] Kyriazanos, I. D. , Sfiniadakis, I. , Gizaris, V. , Hountis, P. , Hatziveis, K. , Dafnopoulou, A. , & Datsakis, K. (2002). The incidence of helicobacter pylori infection is not increased among obese young individuals in Greece. Journal of Clinical Gastroenterology, 34(5), 541–546. 11960066 10.1097/00004836-200205000-00012

[fsn34152-bib-0027] Lane, K. E. , Davies, I. G. , Darabi, Z. , Ghayour‐Mobarhan, M. , Khayyatzadeh, S. S. , & Mazidi, M. (2022). The association between ultra‐processed foods, quality of life and insomnia among adolescent girls in northeastern Iran. International Journal of Environmental Research and Public Health, 19(10), 6338. 35627875 10.3390/ijerph19106338 PMC9141842

[fsn34152-bib-0028] Leo, E. , Martínez, E. , & Segura Campos, M. R. (2020). Effect of ultra‐processed diet on gut microbiota and thus its role in neurodegenerative diseases. Nutrition, 71, 110609. 31837645 10.1016/j.nut.2019.110609

[fsn34152-bib-0029] Malfertheiner, P. , Constanza Camargo, M. , El‐Omar, E. , Liou, J.‐M. , Peek, R. , Schulz, C. , Smith, S. I. , & Suerbaum, S. (2023). Helicobacter pylori infection. Nature Reviews Disease Primers, 9(1), 19. 10.1038/s41572-023-00431-8 PMC11558793 37081005

[fsn34152-bib-0030] Mard, S. A. , Haghighian, H. K. , Sebghatulahi, V. , & Ahmadi, B. (2014). Dietary factors in relation to helicobacter pylori infection. Gastroenterology Research and Practice, 2014, 1–5. 10.1155/2014/826910 PMC4275652 25574164

[fsn34152-bib-0031] Marti, A. , Marcos, A. , & Martinez, J. A. (2001). Obesity and immune function relationships. Obesity Reviews, 2(2), 131–140. 12119664 10.1046/j.1467-789x.2001.00025.x

[fsn34152-bib-0032] Martini, D. , Godos, J. , Bonaccio, M. , Vitaglione, P. , & Grosso, G. (2021). Ultra‐processed foods and nutritional dietary profile: A meta‐analysis of nationally representative samples. Nutrients, 13(10), 3390. 34684391 10.3390/nu13103390 PMC8538030

[fsn34152-bib-0033] Meduri, G. U. (2001). Clinical review: A paradigm shift: The bidirectional effect of inflammation on bacterial growth. Clinical implications for patients with acute respiratory distress syndrome. Critical Care, 6(1), 1–6. 10.1186/cc1450 PMC137394 11940263

[fsn34152-bib-0034] Mezmale, L. , Coelho, L. G. , Bordin, D. , & Leja, M. (2020). Epidemiology of helicobacter pylori. Helicobacter, 25, e12734. 32918344 10.1111/hel.12734

[fsn34152-bib-0035] Mhaskar, R. S. , Ricardo, I. , Azliyati, A. , Laxminarayan, R. , Amol, B. , Santosh, W. , & Boo, K. (2013). Assessment of risk factors of helicobacter pylori infection and peptic ulcer disease. Journal of Global Infectious Diseases, 5(2), 60–67. 23853433 10.4103/0974-777X.112288 PMC3703212

[fsn34152-bib-0036] Monteiro, C. A. , Cannon, G. , Levy, R. B. , Moubarac, J.‐C. , Louzada, M. L. C. , Rauber, F. , Khandpur, N. , Cediel, G. , Neri, D. , & Martinez‐Steele, E. (2019). Ultra‐processed foods: What they are and how to identify them. Public Health Nutrition, 22(5), 936–941. 30744710 10.1017/S1368980018003762 PMC10260459

[fsn34152-bib-0037] Monteiro, C. A. , Cannon, G. , Moubarac, J.‐C. , Levy, R. B. , Louzada, M. L. C. , & Jaime, P. C. (2018). The UN decade of nutrition, the NOVA food classification and the trouble with ultra‐processing. Public Health Nutrition, 21(1), 5–17. 28322183 10.1017/S1368980017000234 PMC10261019

[fsn34152-bib-0038] Mourot, J. , Thouvenot, P. , Charles Couet, J. M. , Antoine, A. K. , & Debry, G. (1988). Relationship between the rate of gastric emptying and glucose and insulin responses to starchy foods in young healthy adults. The American Journal of Clinical Nutrition, 48(4), 1035–1040. 3048076 10.1093/ajcn/48.4.1035

[fsn34152-bib-0039] Nseir, W. , Mograbi, J. , Di Castro, N. , Abu‐Elheja, O. , Abu‐Rahmeh, Z. , Khamaysi, I. , Samara, M. , & Assy, N. (2012). On the association between soft drink consumption and helicobacter pylori infection. Digestive Diseases and Sciences, 57, 981–986. 22057241 10.1007/s10620-011-1963-9

[fsn34152-bib-0040] Oh, B. , Kim, B.‐S. , Kim, J. W. , Kim, J. S. , Koh, S.‐J. , Kim, B. G. , Lee, K. L. , & Chun, J. (2016). The effect of probiotics on gut microbiota during the helicobacter pylori eradication: Randomized controlled trial. Helicobacter, 21(3), 165–174. 26395781 10.1111/hel.12270

[fsn34152-bib-0041] Öztekin, M. , Yılmaz, B. , Ağagündüz, D. , & Capasso, R. (2021). Overview of helicobacter pylori infection: Clinical features, treatment, and nutritional aspects. Diseases, 9(4), 66. 34698140 10.3390/diseases9040066 PMC8544542

[fsn34152-bib-0042] Pasarica, M. , & Dhurandhar, N. V. (2007). Infectobesity: Obesity of infectious origin. Advances in Food and Nutrition Research, 52, 61–102. 17425944 10.1016/S1043-4526(06)52002-9

[fsn34152-bib-0043] Pereira, M. A. , Kartashov, A. I. , Ebbeling, C. B. , Van Horn, L. , Slattery, M. L. , Jacobs Jr, D. R. , & Ludwig, D. S. (2005). Fast‐food habits, weight gain, and insulin resistance (the CARDIA study): 15‐year prospective analysis. The Lancet, 365(9453), 36–42. 10.1016/S0140-6736(04)17663-0 15639678

[fsn34152-bib-0044] Santos, S. , Oliveira, A. , & Lopes, C. (2013). Systematic review of saturated fatty acids on inflammation and circulating levels of adipokines. Nutrition Research, 33(9), 687–695. 24034567 10.1016/j.nutres.2013.07.002

[fsn34152-bib-0045] Schnabel, L. , Buscail, C. , Sabate, J.‐M. , Bouchoucha, M. , Kesse‐Guyot, E. , Alles, B. , Touvier, M. , Monteiro, C. A. , Hercberg, S. , & Benamouzig, R. (2018). Association between ultra‐processed food consumption and functional gastrointestinal disorders: Results from the French NutriNet‐Santé cohort. Official Journal of the American College of Gastroenterology| ACG, 113(8), 1217–1228. 10.1038/s41395-018-0137-1 29904158

[fsn34152-bib-0046] Shu, L. , Zheng, P.‐F. , Zhang, X.‐Y. , & Feng, Y.‐L. (2019). Dietary patterns and helicobacter pylori infection in a group of Chinese adults ages between 45 and 59 years old: An observational study. Medicine, 98(2), e14113. 30633225 10.1097/MD.0000000000014113 PMC6336658

[fsn34152-bib-0047] Sohouli, M. H. , Haghshenas, N. , Pouladi, F. , Sayyari, A. , Olang, B. , Găman, M.‐A. , Kord‐Varkaneh, H. , & Fatahi, S. (2021). Association between glycemic index and helicobacter pylori infection risk among adults: A case‐control study. Nutrition, 83, 111069. 33348108 10.1016/j.nut.2020.111069

[fsn34152-bib-0048] Srour, B. , Fezeu, L. K. , Kesse‐Guyot, E. , Alles, B. , Debras, C. , Druesne‐Pecollo, N. , Chazelas, E. , Deschasaux, M. , Hercberg, S. , & Galan, P. (2020). Ultraprocessed food consumption and risk of type 2 diabetes among participants of the NutriNet‐Santé prospective cohort. JAMA Internal Medicine, 180(2), 283–291. 31841598 10.1001/jamainternmed.2019.5942 PMC6990737

[fsn34152-bib-0049] Srour, B. , Fezeu, L. K. , Kesse‐Guyot, E. , Allès, B. , Méjean, C. , Andrianasolo, R. M. , Chazelas, E. , Deschasaux, M. , Hercberg, S. , & Galan, P. (2019). Ultra‐processed food intake and risk of cardiovascular disease: Prospective cohort study (NutriNet‐Santé). BMJ, 365, l1451. 31142457 10.1136/bmj.l1451 PMC6538975

[fsn34152-bib-0050] Srour, B. , Kordahi, M. C. , Bonazzi, E. , Deschasaux‐Tanguy, M. , Touvier, M. , & Chassaing, B. (2022). Ultra‐processed foods and human health: From epidemiological evidence to mechanistic insights. The Lancet Gastroenterology & Hepatology., 7, 1128–1140. 35952706 10.1016/S2468-1253(22)00169-8

[fsn34152-bib-0051] Suki, M. , Weissman, Y. L. , Boltin, D. , Itskoviz, D. , Perets, T. T. , Comaneshter, D. , Cohen, A. , Niv, Y. , Dotan, I. , & Leibovitzh, H. (2018). Helicobacter pylori infection is positively associated with an increased BMI, irrespective of socioeconomic status and other confounders: A cohort study. European Journal of Gastroenterology & Hepatology, 30(2), 143–148. 29120907 10.1097/MEG.0000000000001014

[fsn34152-bib-0052] Xia, Y. , Meng, G. , Zhang, Q. , Liu, L. , Hongmei, W. , Shi, H. , Bao, X. , Qian, S. , Yeqing, G. , & Fang, L. (2016). Dietary patterns are associated with helicobacter pylori infection in Chinese adults: A cross‐sectional study. Scientific Reports, 6(1), 1–8. 27573193 10.1038/srep32334 PMC5004161

[fsn34152-bib-0053] Xu, C. , Yan, M. , Sun, Y. , Joo, J. , Wan, X. , Chaohui, Y. , Wang, Q. , Shen, C. , Chen, P. , & Li, Y. (2014). Prevalence of helicobacter pylori infection and its relation with body mass index in a Chinese population. Helicobacter, 19(6), 437–442. 25256639 10.1111/hel.12153

[fsn34152-bib-0054] YaoBin, Y.‐O. , YaoBin, Y.‐O. , Yi, H. Y. H. , Yin, Z. Y. Z. , & NongHua, L. N. H. L. (2019). The effect of antioxidants on helicobacter pylori eradication: A systematic review with meta‐analysis. 10.1111/hel.12535 30191635

